# Rapid refeeding in anorexia nervosa: A dialectic balance

**DOI:** 10.1002/eat.23698

**Published:** 2022-03-25

**Authors:** Randolf Staab, Julia Campagna, Julia Ma, Anjana Sengar

**Affiliations:** ^1^ Trillium Health Partners Mississauga Ontario Canada; ^2^ Department of Psychiatry University of Toronto Toronto Ontario Canada; ^3^ Institute for Better Health, Trillium Health Partners Mississauga Ontario Canada

**Keywords:** anorexia nervosa, hypophosphatemia, rapid, refeeding syndrome, weight restoration

## Abstract

**Objective:**

To examine the impact of our new rapid refeeding protocol on patients with anorexia nervosa (AN) in our Eating Disorders Program. We hypothesize that the new protocol would lead to a more rapid weight gain and a shorter length of stay, with no effect on medical complications or program completion.

**Method:**

This cohort design included consecutive inpatients and day hospital patients admitted to the program with a BMI <18 kg/m^2^ and a diagnosis of AN between 2007 and 2020; *N* = 326 patients. Main outcomes measured were rate of weight gain and length of stay. Safety indicators included electrolyte disturbances and supplementation required, complications including refeeding syndrome and completion of the program. A *p* value <.05 was considered statistically significant.

**Results:**

Total length of stay was 21 days shorter for patients on the rapid refeeding protocol compared to the traditional refeeding protocol. Patients on the new protocol gained 0.21 more kg/week compared to patients on the old protocol. There was no difference in completion rates between programs. Electrolyte imbalances were mild to moderate and easily treated with oral electrolyte supplementation. There were no deaths or cases of refeeding syndrome with either protocol.

**Discussion:**

This is the first Canadian study to assess the effectiveness and safety of rapid refeeding in an adult population. Rapid refeeding protocols can be safely administered and are cost effective. Shorter hospital admissions are desirable to minimize possible regression and dependency on inpatient services and positively impacts patients' quality of life.

**Public Significance:**

This study advances the idea that rapid refeeding in patients with anorexia nervosa can be administered safely and effectively with close medical monitoring. In addition, rapid refeeding leads to shorter hospital stays, with a cost‐savings to the health system. Shorter admissions are desirable to minimize possible regression and dependency on inpatient services and also positively impacts patients' quality of life.

## Graphical abstract

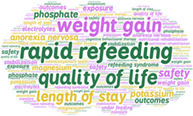

## 1 INTRODUCTION

Anorexia nervosa (AN) is a life threatening, complex psychiatric/medical disorder that has been challenging to treat since it was first described in the medical literature by Sir William Gull in 1873. The often‐enduring nature of the disorder can lead to lengthy treatment that is complex and costly to the medical system. Despite AN's high morbidity and mortality, there is a paucity of high‐quality research in this field. Reasons include its relatively low incidence and prevalence, high cost of providing treatment, high drop‐out rates in studies, and the high degree of denial and resistance commonly seen in patients with AN. A recently published controlled clinical trial addressed the optimal approach to weight restoration in AN along with 1‐year follow‐up of outcomes (Garber et al., 2021; Golden et al., 2021). However, there are no clear evidence‐based recommendations for the core components of treatment of AN: nutritional rehabilitation and refeeding to a healthy weight. Due to the lack of large, randomized controlled studies in the field of Eating Disorders, treatment guidelines are often based on expert opinion, “clinical confidence,” and standards of care based on historical precedent rather than scientific evidence (Redgrave et al., 2015). The lack of a gold standard for refeeding in AN has led to a variety of inconsistent treatment protocols internationally, based on local or regional preferences and biases (Hart et al., 2011; O'Connor & Nicholls, 2013; Schwartz et al., 2008). Moreover, current established refeeding guidelines are often inconsistent, vague, contradictory, and lacking in empirical evidence which can cause confusion for treating clinicians, patients, and families alike (The Royal Colleges of Psychiatrists, Physicians and Pathologists, 2019).

Most traditional, established guidelines in Eating Disorders recommend a very conservative approach to refeeding using a “start low, go slow” approach. For an underweight female weighing 40 kg, the refeeding guidelines on AN from Australia, New Zealand, Europe, the UK NICE, and MARSIPAN all recommend low starting calories of 200–1200 kcal/day or 5–20 kcal/kg with slow, gradual increases to hypothetically prevent complications and refeeding syndrome (American Psychiatric Association, 2006; Association Française Pour le Développement des Approches Spécialisées des Troubles du Comportement Alimentaire, 2010; Association of the Scientific Medical Societies in Germany, 2018; Hay, 2014; Kezelman et al., 2016; National Institute for Health and Care Excellence, 2017; Providence Health Care & British Columbia Ministry of Health, 2012; The Royal Colleges of Psychiatrists, Physicians and Pathologists, 2019). The American Psychiatric Association Treatment Guidelines for Eating Disorders recommend starting calories at only 1000–1600 kcal/day or as low as 600–700 kcal/day in some instances (American Psychiatric Association, 2006). There are no established national Canadian guidelines for Eating Disorders; however, the Provincial Clinical Guidelines for British Columbia suggest starting calories between 800–1000 and 20–25 kcal/day (Providence Health Care & British Columbia Ministry of Health, 2012). Many of these guidelines, which adopt a very cautious approach to refeeding due to fears of refeeding syndrome, are outdated. However, several studies published between 2010 and 2021 have adopted a more rapid refeeding approach to AN with good results (Garber et al., 2012, 2013; Gentile et al., 2010; Golden et al., 2013; Haynos et al., 2016; Koerner et al., 2020; Leclerc et al., 2013; Madden et al., 2015; Parker et al., 2016; Pettersson et al., 2016; Redgrave et al., 2015; Rigaud et al., 2007; Smith et al., 2016; Whitelaw et al., 2010). The treatment of AN needs to balance the urgent need for weight restoration and nutritional rehabilitation while also minimizing the medical risks of refeeding. Several published case reports of refeeding syndrome between 1980 and 2000 were influential in establishing cautious and conservative recommendations for nutritional rehabilitation in AN (Beumont & Large, 1991; Fisher et al., 2000; Kohn et al., 1998; Norris et al., 2012; Weinsier & Krumdieck, 1981). The unpredictable nature and presentation of refeeding syndrome coupled with limited controlled trials have likely perpetuated a cautious approach to refeeding in the field of Eating Disorders.

Refeeding syndrome is a complex, potentially lethal metabolic disorder which can occur during the early stages of refeeding a very malnourished individual. The main biochemical marker is low phosphate, but refeeding syndrome can also lead to low potassium, sodium, magnesium, thiamine, abnormal liver enzymes, hemolytic anemia, high glucose, and abnormalities in white blood cells leading to impaired chemotaxis and abnormal phagocytosis (Society for Adolescent Health and Medicine, 2014). Numerous body systems can be negatively affected leading to neurological, cardiac, and respiratory complications, end‐organ damage, and sudden death within a few days to weeks of increasing caloric intake (Hearing, 2004; Society for Adolescent Health and Medicine, 2014). Refeeding hypophosphatemia and cardiac and neurological events usually develop within the first 7–10 days of nutritional rehabilitation (Matthews et al., 2018; Parker et al., 2016). Refeeding syndrome can be defined by three criteria: (1) severely low electrolytes, (2) peripheral edema or acute circulatory fluid overload, and (3) disturbance to organ function including respiratory failure, cardiac failure, and pulmonary edema (Parker et al., 2016; Rio et al., 2013). Several authors have questioned whether refeeding syndrome is even preventable by caloric manipulation (Garber et al., 2016; Kohn et al., 1998; O'Connor & Goldin, 2011; Society for Adolescent Health and Medicine, 2014). However, the severity of malnutrition pretreatment seems to be a more important risk factor for refeeding hypophosphatemia, rather than the energy intake or rate of weight gain during treatment (Haynos et al., 2016; O'Connor & Nicholls, 2013; Ornstein et al., 2003; Parker et al., 2016; Redgrave et al., 2015). Review papers in the field of Eating Disorders have reported an average incidence of hypophosphatemia of 14%, with observed rates as high as 37%–45% (Garber et al., 2012; Leitner et al., 2016; Norris, 2016; Redgrave et al., 2015; Society for Adolescent Health and Medicine, 2014).

The Intensive Eating Disorders Program is located at the Credit Valley Hospital (CVH) site of Trillium Health Partners, Canada's largest community‐based, academically affiliated hospital system. The program began in 2007 and consists of a four‐bed inpatient unit for severe cases of AN and an intensive Day Hospital Program for eight patients with mild to moderate AN, bulimia nervosa, and other Eating disorders. The program is comprised of an interprofessional team including psychiatrists, psychologists, dieticians, registered nurses, social workers, an occupational therapist, and a diet technician that has specialized training and interest in eating disorders. Patients referred to the program are seen in consultation by a psychiatrist or psychologist and diagnosed with AN including subtype using a structured clinical interview according to the Diagnostic and Statistical Manual of Mental Disorders (DSM) 4 or 5 criteria depending on the year they were assessed. The main goals of treatment are nutritional rehabilitation, normalization of eating and weight gain to at least a BMI of 19.5 kg/m^2^, medical stabilization, cessation of eating disorder symptoms, improving cognition, and skill acquisition. The Eating Disorders Program at CVH is a structured, behavioral, lenient program that is based on best practice guidelines. Treatment is primarily group based with a strong emphasis on Cognitive Behavioral Therapy, Dialectical Behavioral Skill Training (DBT), and contingency management. Patients are supported by nurses around the clock and with video surveillance to promote a healthy milieu that encourages rest and low activity. Patients are medically monitored by program staff with regular vitals, blood work, and ECGs. The inpatient and day programs are integrated, and patients attend meals and therapy groups together throughout the day. Inpatients who are medically stabilized on the inpatient unit graduate to the Day Hospital Program for further treatment. Our Day Hospital Program provides over 30 hours per week of supervised meals, therapy groups, individual and family sessions, skill practice, and homework. After completing our intensive Day Hospital Program, patients can follow‐up in our transition program for gradually tapered treatment and support for up to 5 months. All refeeding in our program was consistent with Canada's Food Guide and done with regular, oral food to promote balance, variety, moderation, and exposure. Meal completion was a mandatory part of treatment; any incomplete portions of meals are replaced with high‐calorie liquid meal supplement. Post meals, patients are closely supervised to prevent purging on the unit.

On the slower traditional refeeding protocol, patients were usually started on 1500–1600 kcal/day and were gradually increased by 300 kcal once a week up to a maintenance level of 2100–2300 kcal using the Harris Benedict Equation with a stress factor of 1.6 to account for higher metabolic needs. This calorie level was maintained as long as patients were gaining greater than 1 kg/week. If patients failed to gain 1 kg, then their prescribed meal plan would increase by 300 kcal/week. Patients in our older protocol were weighed once a week and meal plans were only increased once a week.

Based on emerging evidence for rapid refeeding in AN, our program developed a rapid refeeding protocol in collaboration with the Johns Hopkins Eating Disorders Program in Baltimore, USA. A rapid standardized caloric prescription plan was implemented on January 1, 2017. No other major changes to our program occurred after this time. Studies in adolescents and adults published between 2008 and 2016 using a more rapid approach to refeeding in AN demonstrated more rapid weight gain and a shorter hospital stay without increased risks of electrolyte disturbances or refeeding syndrome (Garber et al., 2013; Golden et al., 2013; Haynos et al., 2016; Koerner et al., 2020; Le Grange, 2013; Madden et al., 2015; Redgrave et al., 2015; Society for Adolescent Health and Medicine, 2015). In our rapid refeeding protocol, patients were started on 1600–1800 kcal/day and were increased by 600 kcal/week to 2800 kcal/day by day 21, regardless of weight gain in the first few weeks. After 2800 kcal/day, if patients failed to gain 1 kg/week their meal plans were increased by another 300–400 kcal/day every week in collaboration with our program dietician.

The primary aims of our study were to examine the effect of our institutional change in refeeding protocol on the rate of weight gain, and length of stay in our inpatient and day program. Secondary aims of the study were to look at safety indicators such as electrolyte disturbances and supplementation, refeeding syndrome, transfer to an internal medical ward, deaths, and completion of the program.

We hypothesized that the change in our refeeding protocol would lead to more rapid weight gain and a shorter length of stay, with no increases in medical complications or program drop‐outs.

## 2 METHODS

**FIGURE 1:**
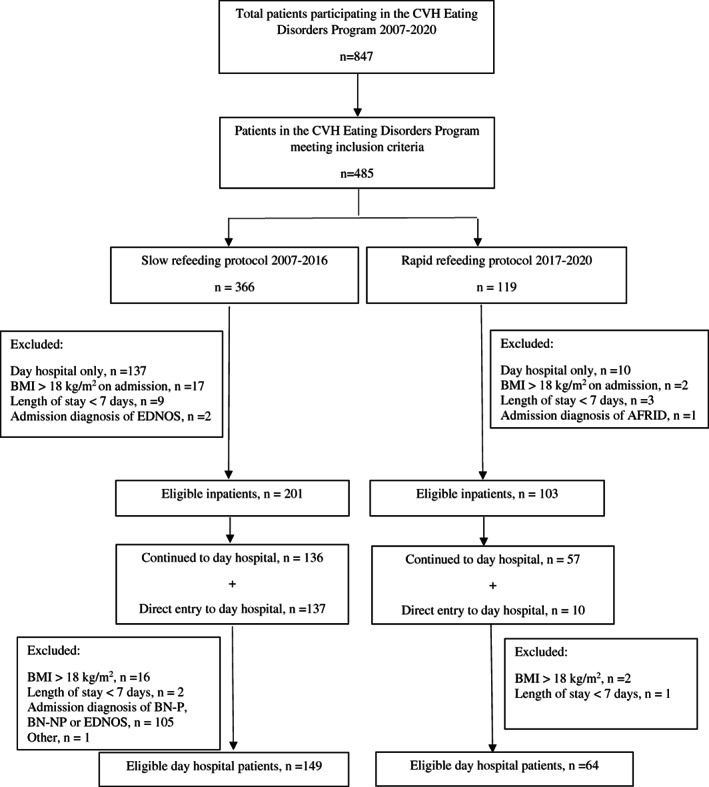
Screening and enrollment

The project was determined by the Research Ethics Board at Trillium Health Partners to not be human subject research requiring REB review and oversight. We followed a natural experimental design comparing patients with AN admitted to our Eating Disorder Program before and after an institutional change in our refeeding protocol. We included all consecutive inpatients and day hospital patients admitted to the CVH Eating Disorders Program with a BMI less than 18 kg/m^2^ and a diagnosis of AN between May 7, 2007 and December 31, 2020. Patients were excluded from the review if they had a diagnosis of Avoidant Restrictive Food Intake Disorder, Eating Disorder Not Otherwise Specified, or bulimia nervosa. Patients were also excluded if their inpatient or day hospital stay was less than 7 days, or if they required naso‐gastric tube feeding or a modified meal plan based on other medical diagnoses (Figure 1).

Data were collected retrospectively for patients admitted on the slow refeeding protocol and obtained prospectively for patients admitted on the rapid refeeding protocol. All inpatients were weighed routinely by nursing staff using a consistent method in a gown, with a medical Healthometer Professional electronic scale. We obtained data on each patient's age, sex, admission and discharge weights, rate of weight gain, number of days on the weight gain protocol, target weight, length of stay, rate of completion, reason for premature discharge, and days until target BMI was achieved.

We obtained measures of inorganic phosphorus, potassium, and magnesium levels for inpatients. For laboratory values, we report total numbers and percentages of those with each of the laboratory abnormalities. Group means, *SD*s, medians, and ranges of these values are reported. Hypophosphatemia was considered present when levels fell below the reference range of 0.87–1.52 mmol/L. We rated hypophosphatemia as mild if 0.65–0.91 mmol/L, moderate if 0.32–0.64 mmol/L, and severe if <0.31 mmol/L (<1.0 mg/dl) (Matthews et al., 2018; Ornstein et al., 2003; Redgrave et al., 2015; Whitelaw et al., 2010). For phosphate, potassium, and magnesium, we recorded the level on admission, the lowest level during their inpatient admission, severity of the low value, number of low values recorded per admission, and number of days from admission that the nadir was reached. Other secondary safety outcome measures were rates and severity of hypokalemia and hypomagnesemia, and the use of phosphate, magnesium, and potassium supplements to treat deficiencies. Hypokalemia was considered mild if 3.1–3.4 mmol/L, moderate if 2.6–3.0 mmol/L, and severe if less than 2.5 mmol/L. Hypomagnesemia was classified as mild/moderate if between 0.51 and 0.70 mmol/L and severe if less than 0.50 mmol/L (Matthews et al., 2018; Parker et al., 2016; Redgrave et al., 2015; Whitelaw et al., 2010). We also examined rates of refeeding syndrome, transfer to a medical ward, and death. For patients admitted more than once during the study period, admissions were treated as independent (Redgrave et al., 2015; Whitelaw et al., 2010) given that weight, rate of gain, length of stay, laboratory values, and risk of refeeding syndrome could change with each subsequent admission of the same patient.

Demographic and clinical data are presented as means and *SD*s, medians and interquartile ranges (IQR), and counts and percentages as appropriate. Q–Q plots and results of Shapiro–Wilk tests were examined to assess the underlying distribution of continuous outcomes. Rate of weight gain was reported as kg/week, and days to reach a target BMI of 19.5 kg/m^2^ was reported as a mean with *SD*. Student's *t*‐tests and Wilcoxon rank sum tests were used to compare continuous outcomes and *χ* ^2^ and Fisher's exact tests were used to compare categorical outcomes. Effect sizes for continuous variables are presented as Cohen's *d* and rank‐biserial correlation, and Cramer's *V* for categorical variables. An alpha level of 0.05 was used for all testing and 95% confidence intervals (CIs) are reported. Linear regression models were used to estimate the effect of the protocol change on the comprehensive (inpatient plus day hospital) total length of stay and the rate of weight gain per week. Estimates were adjusted for admission diagnosis (AN‐restricting subtype vs. AN‐binge/purge). All analyses were conducted in R (Version 3.6.0; R Core Team, 2019).

## 3 RESULTS

A total of 326 inpatients with AN were assessed between 2007 and 2020. Table 1 shows the baseline characteristics of patients who were enrolled in the slow refeeding program from 2007 to 2016 and the new rapid refeeding program from 2017 to 2020. There were no statistically significant differences between the two cohorts.

**TABLE 1:** Baseline characteristics of patients admitted to the inpatient program

	Slow protocol	Rapid protocol
*n* ^a^	201	103
Age, mean (SD)	29.27 (11.80)	28.83 (11.01)
Age group, *n* (%)		
<23	78 (38.8)	34 (33.0)
23–32	63 (31.3)	38 (36.9)
>32	60 (29.9)	31 (30.1)
Sex, *n* (%)		
Female	197 (98.0)	102 (99.0)
Male	4 (2.0)	1 (1.0)
Admission weight (kg), mean (SD)	39.62 (6.13)	39.88 (5.79)
Admission BMI (kg/m^2^), mean (SD)	14.69 (1.70)	14.88 (1.38)
Diagnosis, *n* (%)		
AN‐BP	93 (46.3)	51 (49.5)
AN‐R	108 (53.7)	52 (50.5)

Abbreviations: AN‐BP, anorexia‐binge/purge; AN‐R, anorexia‐restricting subtype; BMI, body mass index.

^a^ Excludes direct to day hospital admits.

When examining the inpatient program, the mean weight and mean BMI at inpatient discharge were not significantly different between patients in either cohort (Table 2). The majority of patients completed the rest of their weight gain in the intensive Day Hospital Program to reach a target BMI of 19.5 kg/m^2^. Although the total weight gain was higher on the slow protocol, patients on our rapid refeeding protocol had a higher rate of weight gain with a mean gain of 1.30 kg/week (*SD* = 0.92) compared to 1.08 kg/week (*SD* = 0.51) in the slow refeeding protocol (*p* = .032). Consequently, the length of stay in hospital was reduced from an average of 53 days among patients in the slow refeeding cohort to an average of 40 days among patients on the rapid refeeding protocol (*p* <.001), a 25% decrease in length of inpatient stay. There were no differences in rate of inpatient program completion between the old and new protocols.

**TABLE 2:** In‐patient program: Patient outcomes

	Slow protocol	Rapid protocol	*p* value	Cohen's *d* (95% CI)
***N***	201	103		
Total weight gain (kg), mean (SD)	10.07 (5.46)	8.84 (4.19)	.031	
Weight gain per week (kg/week), mean (SD)	1.08 (0.51)	1.30 (0.92)	.032	
Weight (kg) at in‐patient discharge, mean (SD)	47.28 (5.61)	47.22 (5.23)	.935	0.009 (−0.228, 0.247)
BMI (kg/m^2^) at in‐patient discharge, mean (SD)	17.59 (1.45)	17.66 (1.27)	.629	−0.056 (−0.294, 0.181)
Length of in‐patient stay (days), mean (SD)	53.02 (24.98)	39.94 (16.73)	<.001	0.581 (0.338, 0.822)
Discharge type, *n* (%)				0.092 **(**0.000, 0.191)^a^
Completed program	145 (72.1)	81 (78.6)	.276	
Discharge against medical advice	28 (13.9)	14 (13.6)		
Administrative discharge	28 (13.9)	8 (7.8)		

Abbreviation: CI, confidence interval.

^a^ Cramers *V* test.

There were no differences in weight gain, BMI at discharge or program completion rates between the day hospital (DH) cohorts (Table 3). Length of DH stay was significantly shorter among patients in the new program compared to the old program with a mean of 44 days versus 52 days (*p* = .006).

**TABLE 3:** Day hospital program: Patient outcomes

	Slow protocol	Rapid protocol	*p* value	Cohen's *d* (95% CI)
*n*	149	64		
Diagnosis, *n* (%)				0.112 **(**0.000, 0.246)^a^
AN‐BP	68 (45.6)	37 (57.8)	.139	
AN‐R	81 (54.4)	27 (42.2)		
Weight (kg) on admission, mean (SD)	48.70 (5.11)	48.16 (4.91)	.468	0.108 (−0.187, 0.402)
BMI (kg/m^2^) on admission, mean (SD)	18.06 (1.14)	18.19 (1.48)	.553	−0.099 (−0.396, 0.196)
Outcomes				
Weight (kg) at discharge, mean (SD)	53.53 (6.05)	52.57 (6.53)	.324	0.155 (−0.142, 0.451)
BMI (kg/m^2^) at discharge, mean (SD)	19.91 (1.15)	19.80 (1.75)	.665	0.077 (−0.219, 0.374)
Length of stay in DH (days), mean (SD)	52.11 (21.44)	44.38 (17.36)	.006	0.381 (0.085, 0.676)
Discharge type, *n* (%)				0.082 **(**0.000, 0.197)^a^
Completed program	112 (75.2)	52 (81.2)	.491	
Withdrew against medical advice	14 (9.4)	6 (9.4)		
Administrative discharge	23 (15.4)	6 (9.4)		

Abbreviations: AN‐BP, anorexia‐binge/purge; AN‐R, anorexia‐restricting subtype; BMI, body mass index; DH, day hospital.

^a^ Cramers *V* test.

Over the comprehensive duration of patients' stay (Inpatient plus Day Hospital Program), we observed a higher total weight gain among patients following the old program (Table 4). This finding was expected since the overall length of stay was longer in the old program. Of the patients who achieved target BMI, target was reached approximately 23 days sooner in the new rapid protocol. However, days to target BMI was only available for 105 patients in the slow refeeding program and 45 patients in the rapid refeeding program.

**TABLE 4:** Unadjusted primary outcomes: Combined inpatient and day hospital

	Slow protocol	Rapid protocol	*p* value	Cohen's *d* (95% CI)
*n*	215	111		
Total weight gain (kg), mean (SD)	9.80 (5.53)	8.59 (4.15)	.043	0.238 (0.007, 0.467)
Rate of weight gain (kg/week), mean (SD)	1.05 (0.53)	1.27 (0.90)	.005	−0.334 (−0.564, −0.103)
Total length of stay (days), mean (SD)	85.69 (44.67)	62.65 (30.40)	<.001	0.570 (0.338, 0.803)
Days to target BMI (19.5 kg/m^2^), mean (SD), *n*	81.61 (30.20), 105	59.04 (24.52), 45	<.001	0.799 (0.438, 1.158)

Abbreviations: BMI, body mass index; CI, confidence interval.

Following adjustment for admission diagnosis, on average, the total length of stay was 22 days shorter among patients in the new protocol and these patients gained 0.21 more kg/week in the new program compared to the old program (Table 5).

**TABLE 5:** Primary outcomes: regression analysis

	Total length of stay (days)				Rate of weight gain (kg/week)			
Variable	Estimate	*SE*	*p* value	95% CI	Estimate	*SE*	*p* value	95% CI
Rapid versus slow refeeding	−22.20	4.64	<.001	(−31.33, −13.07)	0.21	0.078	.007	(0.06, 0.37)
AN‐R versus AN‐BP	15.97	4.40	<.001	(7.31, 24.62)	−0.24	0.074	.001	(−0.38, 0.09)

Abbreviations: AN‐BP, anorexia‐binge/purge; AN‐R, anorexia‐restricting subtype; CI, confidence interval.

Hypophosphatemia was not significantly different between the slow and rapid refeeding protocols after admission (Table 6). There were no severe cases of hypophosphatemia in either the slow or rapid protocols. Total cases of mild and moderate hypophosphatemia were not significantly different between the groups. The median number of days until the lowest phosphate level was recorded was on Day 2 in old cohort and on Day 3 in the new.

**TABLE 6:** Post admission electrolyte blood plasma levels

Measure	Slow protocol	Rapid protocol	*p* value	Cramer's *V* (95% CI)
*Phosphate* (PO^4^)				
Hypophosphatemia at any point after admission				
Yes	29 (14.4)	23 (22.3)	.116	0.099 (0.000, 0.211)
No	172 (85.6)	80 (77.7)		
Phosphate level^a^ if low after admission, median [IQR]	0.83 [0.74, 0.86]	0.78 [0.70, 0.85]	.455	0.121 (−0.194, 0.414)^b^
Severity of hypophosphatemia after admission				
Mild	26 (89.7)	20 (87.0)	1.000	0.042 (0.000, 0.298)
Moderate	3 (10.3)	3 (13.0)		
Magnesium (Mg)				
Hypomagnesemia at any point after admission				
Yes	94 ( 46.8)	17 (16.5)	<.001	0.297 (0.185, 0.409)
No	107 (53.2)	86 (83.5)		
Magnesium level^a^ if low after admission, median [IQR]	0.66 [0.64, 0.68]	0.67 [0.66, 0.70]	.063	−0.282 (−0.529, 0.009)^b^
Severity of hypomagnesemia after admission				
Mild/moderate	93 (98.9)	16 (94.1)	.284	0.130 (0.000, 0.316)
Severe	1 (1.1)	1 (5.9)		
Potassium (K)				
Hypokalemia at any point after admission				
Yes	10 (5.0)	26 (25.2)	<.001	0.297 (0.184, 0.409)
No	191 (95.0)	77 (74.8)		
Potassium level^a^ if low after admission, median [IQR]	3.35 [3.30, 3.40]	3.35 [3.20, 3.40]	.717	0.073 (−0.339, 0.462)^b^
Severity of hypokalemia after admission				
Mild	9 (90.0)	25 (96.2)	.484	0.120 (0.000, 0.446)
Moderate	1 (10.0)	1 (3.8)		

Abbreviations: CI, confidence interval; IQR, interquartile range.

^a^ Blood values are listed in mmol/L.

^b^ *r* rank biserial.

There were more low magnesium values after admission in our older, slower refeeding protocol than the new rapid protocol; however, this was not statistically different. The severity of the hypomagnesemia did not differ, and the vast majority were mild/moderate cases.

There were more low potassium levels recorded at any point after admission with the new rapid protocol compared to the old protocol. This finding may in part be due to the fact that more patients entered the program with low admission potassium levels. However, the severity of low potassium did not differ between groups after admission (Table 6), and the vast majority were of mild severity treated with oral supplementation (Table 7).

**TABLE 7:** Electrolyte supplementation

	Slow protocol	Rapid protocol	*p* value	*r* rank biserial (95% CI)
Received phosphate supplement, *n*	21	13		
Phosphate dose (mmol/day), median [IQR]	17.60 [16.00, 22.90]	16.00 [0.00, 16.00]	.009	0.524 (0.175, 0.756)
Received magnesium supplement, *n*	48	7		
Magnesium dose (mg/day), median [IQR]	00.00 [0.00, 270.28]	0.00 [0.00, 150.00]	.503	0.143 (−0.308, 0.541)
Received potassium supplement, *n*	31	29		
Potassium dose (meq/day), median [IQR]	30.00 [20.00, 45.00]	20.00 [20.00, 29.40]	.055	0.283 (−0.004, 0.526)

Abbreviation: CI, confidence interval; IQR, interquartile range.

Among patients who experienced low magnesium and potassium levels after admission, the median number of days to nadir was not different between the traditional and rapid programs across all three electrolyte groups.

The amounts of phosphate supplements used to treat phosphate deficiencies were higher in the slow protocol compared to the rapid protocol. The amounts of potassium and magnesium supplements used did not differ between cohorts (Table 7). An admission BMI less than 14 kg/m^2^ was associated with a 2.76 times higher odds of experiencing low phosphate after admission compared to patients with a BMI greater than/equal to 14 kg/m^2^.

There were no deaths or cases of refeeding syndrome that met all three diagnostic criteria (Rio et al., 2013) with either protocol over the 14‐year period. Four patients on the slow refeeding protocol and one patient on the rapid refeeding protocol were transferred from our Eating Disorders unit to a medical ward for medical instability not related to refeeding.

## 4 DISCUSSION

This is the first publication to assess the effectiveness and the safety of rapid refeeding in an adult population in Canada. This study adds to the growing body of literature demonstrating that rapid refeeding protocols can be safely administered, are cost effective, and preferable in many ways (Garber, 2020; Haynos et al., 2016; Redgrave et al., 2015).

This study provides evidence that more rapid weight gain is possible in AN, using a rate of refeeding and starting calories that is higher than what is currently recommended by most international treatment guidelines for AN and higher than what is currently recommended by traditional Eating Disorders Programs in Canada. (American Psychiatric Association, 2006; Association Française pour le Développement des Approches Spécialisées des Troubles du Comportement Alimentaire, 2010; Association of the Scientific Medical Societies in Germany, 2018; MARSIPAN: Management of Really Sick Patients with Anorexia Nervosa, 2018; National Institute for Health and Care Excellence, 2017; Providence Health Care & British Columbia Ministry of Health, 2012; Royal Australian and New Zealand College of Psychiatrists, 2014). This study in combination with others over the last 8 years challenge the “start low, go slow” paradigm of refeeding in AN. There has been increased recognition over the last 5 years that slow, “underfeeding” in AN treatment can have detrimental effects on weight gain, cognition, blood sugars, organ damage, cardiac abnormalities, early weight loss in treatment, and hospitalization, contributing to a worse outcome (Garber et al., 2016; Koerner et al., 2020). Given that a slower rate of weight gain in hospital and day program treatment has been associated with a worse outcome in AN (Castro et al., 2004; Chatelet et al., 2020; Garber et al., 2016; Golden et al., 2013; Lund et al., 2009; Smith et al., 2016; Steinhausen et al., 2008; Zipfel et al., 2000), it is imperative that patients are given enough calories to efficiently restore both physical and mental health in a timely manner (Golden et al., 2013; Lund et al., 2009; Sly & Bamford, 2011; Steinhausen et al., 2008). Our traditional, slow refeeding protocol often resulted in weight loss in the first 1–2 weeks of treatment, because initial calorie prescriptions were typically below resting energy requirements (Garber et al., 2012; Kohn et al., 2011). Patients with AN become hypermetabolic during the refeeding process, which can substantially increase calorie requirements and hinder weight gain (Garber et al., 2012; Pettersson et al., 2016). A faster rate of weight gain in treatment is desirable to rapidly improve cognitive functioning so that patients are more able to engage in meaningful psychotherapy, CBT, and DBT skills, which are essential for a durable, lasting recovery. Even the best therapies will not be effective in the face of malnutrition, low blood sugars, and cognitive impairment. Our rapid refeeding protocol allowed our dietician to introduce more variety into our patients' meal plans earlier in treatment, allowing for more exposure and desensitization to challenging “risk” foods on their hierarchy.

Our rapid refeeding protocol resulted in a decreased overall length of stay of 21 days in patients who completed the entire program using the rapid refeeding protocol. Length of inpatient stay decreased from an average of 53 to 40 days, and in Day Hospital from 52 to 44 days, an overall decrease of 25% and 15%, respectively. Given the limited resources and long waiting lists for intensive Eating Disorder services, the decrease in length of stay is both statistically and clinically significant. The cost to treat AN is among the highest of all psychiatric disorders due to the extensive use of hospital services (Bulik et al., 2007). A 13‐day decrease in length of inpatient stay amounts to a cost savings of $7748 per patient, and would allow our four bed inpatient unit to treat an extra 10–13 patients per year and decrease our waiting list. Over the 9 years of utilizing the slow refeeding protocol, the program averaged 26.7 admissions per year. The new rapid refeeding program averaged 40 admissions per year; providing care to an additional 13.2 patients per year. A shorter hospital admission is further desirable to minimize possible regression and dependency on inpatient services, and to achieve more skill generalization in the patients' usual home environment. A shorter stay also positively impacts the patients' quality of life by minimizing disruption to a patient's school, work, family, and social life (Castro et al., 2004).

The rapid refeeding protocol was well tolerated by patients, as the rate of program completion was not significantly different before and after our institutional change in refeeding practice. Our findings are consistent with other refeeding studies demonstrating the safety of rapid protocols. Among 103 patients over 4 years, there were no full cases of refeeding syndrome, severe hypophosphatemia, or death. One patient was transferred to a medical ward, which was not related to refeeding. Of the patients on our rapid protocol, 22.3% had a low phosphate level sometime after admission, which was not significantly different from our traditional slow refeeding protocol (14.4%) and similar to the findings by Redgrave et al. (2015) of 18.5%. Early detection and correction of electrolyte abnormalities with oral supplements can easily prevent the development of full refeeding syndrome as demonstrated in our study. When low levels of phosphate, potassium, and magnesium occurred in our study, they were usually mild in severity and easily treated with oral supplements.

We believe that concerns of refeeding syndrome and refeeding hypophosphatemia have been overstated. Over 18 published studies to date have used a rapid refeeding approach to varying degrees in adults and adolescents with a total sample size of 1632. Of these studies, none have reported cases of refeeding syndrome or deaths due to refeeding (Agostino et al., 2013; Garber, 2020; Garber et al., 2012, 2013; Gaudiani et al., 2012; Golden et al., 2013; Haynos et al., 2016; Koerner et al., 2020; Leclerc et al., 2013; Madden et al., 2015; Matthews et al., 2018; Parker et al., 2016; Pettersson et al., 2016; Redgrave et al., 2015; Rigaud et al., 2007; Smith et al., 2016; Whitelaw et al., 2010). In fact, most case reports of refeeding syndrome in the literature have occurred in patients with AN who were treated with a slow, traditional, refeeding protocol (Kohn et al., 1998; O'Connor & Goldin, 2011). Increased rates of refeeding hypophosphatemia are associated with lower BMI and more severe emaciation at admission, but not with the rate of refeeding or rates of weight gain (Garber et al., 2013, 2016; Leitner et al., 2016; Ornstein et al., 2003; Parker et al., 2016; Redgrave et al., 2015; Society for Adolescent Health and Medicine, 2014). Our findings are consistent with these literature as an admission BMI <14 kg/m^2^ was associated with a 2.73 times higher odds of experiencing low phosphate after admission compare to patients with an inpatient BMI ≥14 kg/m^2^.

Strengths of our study include a fairly large sample size, including a large proportion of patients who were very malnourished at admission (BMI 14.70–14.98). Given the paucity of randomized, controlled trials in refeeding among patients with AN, the quasi‐experimental design is a strength as it compares higher and lower calorie refeeding in the same program using historical controls. The CVH Eating Disorders Program was relatively stable throughout the 14 years of the study with consistent rules and expectations around eating and meal completion, contingency management, groups, staff, and environment.

There are several limitations to this study. This was a single‐sited nonrandomized controlled study and some data points were collected retrospectively, which relied on complete data being present in patient charts. We collected and analyzed laboratory values for inpatient records only; laboratory data were insufficient to make meaningful comparisons or conclusions for day hospital patients. Data on the number of days to reach the target BMI of 19.5 kg/m^2^ were limited to 105 patients in the old program and 45 patients in the new, as a significant number of patients attended a residential or day program closer to their home to complete weight restoration and recovery. Although significant associations between the slow and rapid refeeding programs and two primary outcomes were noted, we also observed low precision around the estimates, which may suggest the need for a larger sample. We did not examine the long‐term outcomes for patients, which would be important to look at in future studies. Finally, there is risk of maturation bias, as practices evolve over the passage of time and may influence the study outcomes. There were no intentional changes made to our program and psychotherapies during this time.

Historically, Eating Disorder clinicians would endeavor to strike a balance between the benefits of rapid weight gain and medical stabilization against the risk of refeeding syndrome. Given that the risk of refeeding syndrome is rare and was not present in over 18 published studies involving over 1632 patients using a more rapid protocol, it is time that the pendulum swings to a more rapid, assertive approach to refeeding (Golden et al., 2013). Our analysis suggests that initial calorie prescriptions and increases can safely exceed the current recommended guidelines and concerns of refeeding syndrome and refeeding hypophosphatemia are likely overstated. More aggressive refeeding protocols should be implemented with close medical monitoring and prompt treatment of electrolyte abnormalities with supplements to mitigate any potential risks of refeeding syndrome. Given that faster weight gain in both inpatient and outpatient ambulatory settings has been associated with higher weight at discharge and similar outcomes at 1‐year follow‐up (Chatelet et al., 2020; Garber et al., 2016, 2021), it is important to speed up the process of nutritional rehabilitation while patients are in a safe and supervised environment. Shorter hospital admission followed by intensive residential and day hospital treatments have not been found to be detrimental to long‐term outcomes in AN (Smith et al., 2016). Our study adds to a growing body of literature that argues that the risks of underfeeding syndrome are greater and more common than refeeding syndrome in specialized settings for the treatment of AN.

## CONFLICT OF INTEREST

The authors declare no potential conflict of interest.

## Data Availability

Data are available within the article or its supplementary materials. Additional data available on request from the corresponding author.

## References

[eat23698-bib-0001] Agostino, H. , Erdstein, J. , & Di Meglio, G. (2013). Shifting paradigms: Continuous nasogastric feeding with high caloric intakes in anorexia nervosa. The Journal of Adolescent Health: Official Publication of the Society for Adolescent Medicine, 53(5), 590–594. 10.1016/j.jadohealth.2013.06.005 23871800

[eat23698-bib-0003] American Psychiatric Association . (2006). Practice guideline for the treatment of patients with eating disorders (3rd ed.). Arlington, VA: American Psychiatric Association. http://psychiatryonline.org/pb/assets/raw/sitewide/practice_guidelines/guidelines/eatingdisorders.pdf

[eat23698-bib-0004] Association Française pour le Développement des Approches Spécialisées des Troubles du Comportement Alimentaire (2010). Clinical practice guidelines anorexia nervosa: Management. Saint‐Denis, France: Haute Autorité de Santé. https://www.has-sante.fr/jcms/c_1546479/en/guidelines-anorexia-nervosa-management

[eat23698-bib-0005] Association of the Scientific Medical Societies in Germany (2018). Joint German guideline “diagnosis and treatment of eating disorders”. AWMF online. https://www.awmf.org/fileadmin/user_upload/Leitlinien/051_D-Ges_Psychosom_Med_u_aerztliche_Psychotherapie/051-026e_S3_eating-disorders-diagnosis-treatment_2020-07.pdf

[eat23698-bib-0006] Beumont, P. J. , & Large, M. (1991). Hypophosphataemia, delirium and cardiac arrhythmia in anorexia nervosa. The Medical Journal of Australia, 155(8), 519–522. (13) 1943930 10.5694/j.1326-5377.1991.tb93887.x

[eat23698-bib-0007] Bulik, C. M. , Berkman, N. D. , Brownley, K. A. , Sedway, J. A. , & Lohr, K. N. (2007). Anorexia nervosa treatment: A systematic review of randomized controlled trials. The International Journal of Eating Disorders, 40(4), 310–320. 10.1002/eat.20367 17370290

[eat23698-bib-0008] Castro, J. , Gila, A. , Puig, J. , Rodriguez, S. , & Toro, J. (2004). Predictors of rehospitalization after total weight recovery in adolescents with anorexia nervosa. The International Journal of Eating Disorders, 36(1), 22–30. 10.1002/eat.20009 15185268

[eat23698-bib-0009] Chatelet, S. , Wang, J. , Gjoertz, M. , Lier, F. , Monney Chaubert, C. , & Ambresin, A. E. (2020). Factors associated with weight gain in anorexia nervosa inpatients. Eating and Weight Disorders: EWD, 25(4), 939–950. 10.1007/s40519-019-00709-5 (25) 31119585

[eat23698-bib-0010] R Core Team . (2019). R: A language and environment for statistical computing. The R Project for Statistical Computing. https://www.r-project.org/

[eat23698-bib-0011] Fisher, M. , Simpser, E. , & Schneider, M. (2000). Hypophosphatemia secondary to oral refeeding in anorexia nervosa. The International Journal of Eating Disorders, 28(2), 181–187. 10.1002/1098-108x(200009)28:2<181::aid-eat7>3.0.co;2-k 10897080

[eat23698-bib-0012] Garber, A. K. , Cheng, J. , Accurso, E. , Adams, S. , Buckelew, S. , Kapphahn, C. , Kreiter, A. , Le Grange, D. , Machen, V. , Moscicki, A. , Sy, A. , Wilson, L. , & Golden, N. (2021). Short‐term outcomes of the study of refeeding to optimize inpatient gains for patients with anorexia nervosa. Journal of American Medical Association Pediatrics, 175(1), 19. 10.1001/jamapediatrics.2020.3359 PMC7573797 33074282

[eat23698-bib-0013] Garber, A. K. , Mauldin, K. , Michihata, N. , Buckelew, S. M. , Shafer, M. A. , & Moscicki, A. B. (2013). Higher calorie diets increase rate of weight gain and shorten hospital stay in hospitalized adolescents with anorexia nervosa. The Journal of Adolescent Health: Official publication of the Society for Adolescent Medicine, 53(5), 579–584. 10.1016/j.jadohealth.2013.07.014 24054812 PMC4452504

[eat23698-bib-0014] Garber, A. K. , Michihata, N. , Hetnal, K. , Shafer, M. A. , & Moscicki, A. B. (2012). A prospective examination of weight gain in hospitalized adolescents with anorexia nervosa on a recommended refeeding protocol. The Journal of Adolescent Health: Official Publication of the Society for Adolescent Medicine, 50(1), 24–29. 10.1016/j.jadohealth.2011.06.011 22188830 PMC4467563

[eat23698-bib-0015] Garber, A. K. , Sawyer, S. M. , Golden, N. H. , Guarda, A. S. , Katzman, D. K. , Kohn, M. R. , Le Grange, D. , Madden, S. , Whitelaw, M. , & Redgrave, G. W. (2016). A systematic review of approaches to refeeding in patients with anorexia nervosa. The International Journal of Eating Disorders, 49(3), 293–310. 10.1002/eat.22482 26661289 PMC6193754

[eat23698-bib-0016] Gaudiani, J. L. , Sabel, A. L. , Mascolo, M. , & Mehler, P. S. (2012). Severe anorexia nervosa: outcomes from a medical stabilization unit. The International Journal of Eating Disorders, 45(1), 85–92. 10.1002/eat.20889 22170021

[eat23698-bib-0017] Gentile, M. G. , Pastorelli, P. , Ciceri, R. , Manna, G. M. , & Collimedaglia, S. (2010). Specialized refeeding treatment for anorexia nervosa patients suffering from extreme undernutrition. Clinical Nutrition, 29(5), 627–632. 10.1016/j.clnu.2010.03.008 20416994

[eat23698-bib-0018] Golden, N. H. , Cheng, J. , Kapphahn, C. J. , Buckelew, S. M. , Machen, V. I. , Kreiter, A. , Accurso, E. C. , Adams, S. H. , Le Grange, D. , Moscicki, A. B. , Sy, A. F. , Wilson, L. , & Garber, A. K. (2021). Higher‐calorie refeeding in anorexia nervosa: 1‐year outcomes from a randomized controlled trial. Pediatrics, 147(4), e2020037135. 10.1542/peds.2020-037135 33753542 PMC8015147

[eat23698-bib-0019] Golden, N. H. , Keane‐Miller, C. , Sainani, K. L. , & Kapphahn, C. J. (2013). Higher caloric intake in hospitalized adolescents with anorexia nervosa is associated with reduced length of stay and no increased rate of refeeding syndrome. The Journal of Adolescent Health: Official Publication of the Society for Adolescent Medicine, 53(5), 573–578. 10.1016/j.jadohealth.2013.05.014 23830088

[eat23698-bib-0020] Hart, S. , Abraham, S. , Franklin, R. , & Russell, J. (2011). Weight changes during inpatient refeeding of underweight eating disorder patients. European Eating Disorders Review: the Journal of the Eating Disorders Association, 19(5), 390–397. 10.1002/erv.1052 24081714

[eat23698-bib-0021] Hay, P. , Chinn, D. , Forbes, D. , Madden, S. , Newton, R. , Sugenor, L. , Touyz, S. , Ward, W. , & Royal Australian and New Zealand College of Psychiatrists . (2014). Royal Australian and New Zealand College of Psychiatrists clinical practice guidelines for the treatment of eating disorders. The Australian and New Zealand Journal of Psychiatry, 48(11), 977–1008. 10.1177/0004867414555814 25351912

[eat23698-bib-0022] Haynos, A. F. , Snipes, C. , Guarda, A. , Mayer, L. E. , & Attia, E. (2016). Comparison of standardized versus individualized caloric prescriptions in the nutritional rehabilitation of inpatients with anorexia nervosa. The International Journal of Eating Disorders, 49(1), 50–58. 10.1002/eat.22469 26769581 PMC4717916

[eat23698-bib-0023] Hearing, S. D. (2004). Refeeding syndrome. BMJ, 328(7445), 908–909. 10.1136/bmj.328.7445.908 15087326 PMC390152

[eat23698-bib-0025] Kezelman, S. , Rhodes, P. , Hunt, C. , Anderson, G. , Clarke, S. , Crosby, R. D. , & Touyz, S. (2016). Adolescent patients' perspectives on rapid‐refeeding: A prospective qualitative study of an inpatient population. Advances in Eating Disorders, 4(3), 277–292. 10.1080/21662630.2016.1202124

[eat23698-bib-0026] Koerner, T. , Haas, V. , Heese, J. , Karacic, M. , Ngo, E. , Correll, C. U. , Voderholzer, U. , & Cuntz, U. (2020). Outcomes of an accelerated inpatient refeeding protocol in 103 extremely underweight adults with anorexia nervosa at a specialized clinic in Prien, Germany. Journal of Clinical Medicine, 9(5), 1535. 10.3390/jcm9051535 PMC7291118 32438760

[eat23698-bib-0027] Kohn, M. R. , Golden, N. H. , & Shenker, I. R. (1998). Cardiac arrest and delirium: Presentations of the refeeding syndrome in severely malnourished adolescents with anorexia nervosa. The Journal of Adolescent Health: Official Publication of the Society for Adolescent Medicine, 22(3), 239–243. 10.1016/S1054-139X(97)00163-8 9502012

[eat23698-bib-0028] Kohn, M. R. , Madden, S. , & Clarke, S. D. (2011). Refeeding in anorexia nervosa: Increased safety and efficiency through understanding the pathophysiology of protein calorie malnutrition. Current Opinion in Pediatrics, 23(4), 390–394. 10.1097/MOP.0b013e3283487591 21670680

[eat23698-bib-0029] Le Grange, D. (2013). Examining refeeding protocols for adolescents with anorexia nervosa (again): Challenges to current practices. The Journal of Adolescent Health: Official Publication of the Society for Adolescent Medicine, 53(5), 555–556. 10.1016/j.jadohealth.2013.08.015 24138761

[eat23698-bib-0030] Leclerc, A. , Turrini, T. , Sherwood, K. , & Katzman, D. K. (2013). Evaluation of a nutrition rehabilitation protocol in hospitalized adolescents with restrictive eating disorders. The Journal of Adolescent Health: Official Publication of the Society for Adolescent Medicine, 53(5), 585–589. 10.1016/j.jadohealth.2013.06.001 23891242

[eat23698-bib-0031] Leitner, M. , Burstein, B. , & Agostino, H. (2016). Prophylactic phosphate supplementation for the inpatient treatment of restrictive eating disorders. The Journal of Adolescent Health: Official Publication of the Society for Adolescent Medicine, 58(6), 616–620. 10.1016/j.jadohealth.2015.12.001 26774639

[eat23698-bib-0032] Lund, B. C. , Hernandez, E. R. , Yates, W. R. , Mitchell, J. R. , McKee, P. A. , & Johnson, C. L. (2009). Rate of inpatient weight restoration predicts outcome in anorexia nervosa. The International Journal of Eating Disorders, 42(4), 301–305. 10.1002/eat.20634 19107835

[eat23698-bib-0033] Madden, S. , Miskovic‐Wheatley, J. , Clarke, S. , Touyz, S. , Hay, P. , & Kohn, M. R. (2015). Outcomes of a rapid refeeding protocol in adolescent anorexia nervosa. Journal of Eating Disorders, 3, 8. 10.1186/s40337-015-0047-1 25830024 PMC4379764

[eat23698-bib-0034] Matthews, K. , Hill, J. , Jeffrey, S. , Patterson, S. , Davis, A. , Ward, W. , Palmer, M. , & Capra, S. (2018). A higher‐calorie refeeding protocol does not increase adverse outcomes in adult patients with eating disorders. Journal of the Academy of Nutrition and Dietetics, 118(8), 1450–1463. 10.1016/j.jand.2018.01.023 29656932

[eat23698-bib-0035] National Institute for Health and Care Excellence . (2017). *Eating disorders: Recognition and treatment* [NICE Guideline No. 69]. https://www.nice.org.uk/guidance/ng69/resources/eating-disorders-recognition-and-treatment-pdf-1837582159813 28654225

[eat23698-bib-0036] Norris, M. L. (2016). Phosphate supplementation during refeeding of hospitalized adolescents with anorexia nervosa‐watch and wait or empirically treat. The Journal of Adolescent Health: Official Publication of the Society for Adolescent Medicine, 58(6), 593–594. 10.1016/j.jadohealth.2016.03.030 27210006

[eat23698-bib-0037] Norris, M. L. , Pinhas, L. , Nadeau, P. O. , & Katzman, D. K. (2012). Delirium and refeeding syndrome in anorexia nervosa. The International Journal of Eating Disorders, 45(3), 439–442. 10.1002/eat.20963 22009708

[eat23698-bib-0038] O'Connor, G. , & Goldin, J. (2011). The refeeding syndrome and glucose load. The International Journal of Eating Disorders, 44(2), 182–185. 10.1002/eat.20791 20127933

[eat23698-bib-0039] O'Connor, G. , & Nicholls, D. (2013). Refeeding hypophosphatemia in adolescents with anorexia nervosa: A systematic review. Nutrition in Clinical Practice: Official Publication of the American Society for Parenteral and Enteral Nutrition, 28(3), 358–364. 10.1177/0884533613476892 23459608 PMC4108292

[eat23698-bib-0040] Ornstein, R. M. , Golden, N. H. , Jacobson, M. S. , & Shenker, I. R. (2003). Hypophosphatemia during nutritional rehabilitation in anorexia nervosa: Implications for refeeding and monitoring. The Journal of Adolescent Health: Official Publication of the Society for Adolescent Medicine, 32(1), 83–88. 10.1016/s1054-139x(02)00456-1 12507806

[eat23698-bib-0041] Parker, E. K. , Faruquie, S. S. , Anderson, G. , Gomes, L. , Kennedy, A. , Wearne, C. M. , Kohn, M. R. , & Clarke, S. D. (2016). Higher caloric refeeding is safe in hospitalised adolescent patients with restrictive eating disorders. Journal of Nutrition and Metabolism, 2016, 5168978. 10.1155/2016/5168978 27293884 PMC4880718

[eat23698-bib-0042] Pettersson, C. , Tubic, B. , Svedlund, A. , Magnusson, P. , Ellegård, L. , Swolin‐Eide, D. , & Forslund, H. B. (2016). Description of an intensive nutrition therapy in hospitalized adolescents with anorexia nervosa. Eating Behaviors, 21, 172–178. 10.1016/j.eatbeh.2016.03.014 26970731

[eat23698-bib-0043] Providence Health Care & British Columbia Ministry of Health (2012). Clinical practice guidelines for the BC eating disorders continuum of services. British Columbia Ministry of Health. http://www.bcchildrens.ca/mental‐health‐services‐site/Documents/Clinical%20Practice%20Guidelines%20for%20the%20BC%20Eating%20Disorders%20Continuum%20of%20Services.pdf

[eat23698-bib-0044] Redgrave, G. W. , Coughlin, J. W. , Schreyer, C. C. , Martin, L. M. , Leonpacher, A. K. , Seide, M. , Verdi, A. M. , Pletch, A. , & Guarda, A. S. (2015). Refeeding and weight restoration outcomes in anorexia nervosa: Challenging current guidelines. The International Journal of Eating Disorders, 48(7), 866–873. 10.1002/eat.22390 25625572

[eat23698-bib-0045] Rigaud, D. , Brondel, L. , Poupard, A. T. , Talonneau, I. , & Brun, J. M. (2007). A randomized trial on the efficacy of a 2‐month tube feeding regimen in anorexia nervosa: A 1‐year follow‐up study. Clinical Nutrition, 26(4), 421–429. 10.1016/j.clnu.2007.03.012 17499892

[eat23698-bib-0046] Rio, A. , Whelan, K. , Goff, L. , Reidlinger, D. P. , & Smeeton, N. (2013). Occurrence of refeeding syndrome in adults started on artificial nutrition support: Prospective cohort study. BMJ Open, 3(1), e002173. 10.1136/bmjopen-2012-002173 PMC3549252 23315514

[eat23698-bib-0047] The Royal Colleges of Psychiatrists, Physicians and Pathologists . (2019). MARSIPAN: Management of Really Sick Patients with Anorexia Nervosa 2nd edition. 10.1192/bja.2017.2

[eat23698-bib-0048] Schwartz, B. I. , Mansbach, J. M. , Marion, J. G. , Katzman, D. K. , & Forman, S. F. (2008). Variations in admission practices for adolescents with anorexia nervosa: A north American sample. The Journal of Adolescent Health: Official Publication of the Society for Adolescent Medicine, 43(5), 425–431. 10.1016/j.jadohealth.2008.04.010 18848669

[eat23698-bib-0049] Sly, R. , & Bamford, B. (2011). Why are we waiting? The relationship between low admission weight and end of treatment weight outcomes. European Eating Disorders Review: The Journal of the Eating Disorders Association, 19(5), 407–410. 10.1002/erv.1061 24081716

[eat23698-bib-0050] Smith, K. , Lesser, J. , Brandenburg, B. , Lesser, A. , Cici, J. , Juenneman, R. , Beadle, A. , Eckhardt, S. , Lantz, E. , Lock, J. , & Le Grange, D. (2016). Outcomes of an inpatient refeeding protocol in youth with anorexia nervosa and atypical anorexia nervosa at children's hospitals and clinics of Minnesota. Journal of Eating Disorders, 4, 35. 10.1186/s40337-016-0124-0 28018595 PMC5165845

[eat23698-bib-0051] Society for Adolescent Health and Medicine . (2014). Refeeding hypophosphatemia in hospitalized adolescents with anorexia nervosa: A position statement of the Society for Adolescent Health and Medicine. The Journal of Adolescent Health: Official Publication of the Society for Adolescent Medicine, 55(3), 455–457. 10.1016/j.jadohealth.2014.06.010 25151056 PMC6159900

[eat23698-bib-0052] Society for Adolescent Health and Medicine . (2015). Position paper of the Society for Adolescent Health and Medicine: Medical management of restrictive eating disorders in adolescents and young adults. The Journal of Adolescent Health: Official Publication of the Society for Adolescent Medicine, 56(1), 121–125. 10.1016/j.jadohealth.2014.10.259 25530605

[eat23698-bib-0053] Steinhausen, H. C. , Grigoroiu‐Serbanescu, M. , Boyadjieva, S. , Neumärker, K. J. , & Winkler Metzke, C. (2008). Course and predictors of rehospitalization in adolescent anorexia nervosa in a multisite study. The International Journal of Eating Disorders, 41(1), 29–36. 10.1002/eat.20414 17647278

[eat23698-bib-0054] Weinsier, R. L. , & Krumdieck, C. L. (1981). Death resulting from overzealous total parenteral nutrition: The refeeding syndrome revisited. The American Journal of Clinical Nutrition, 34(3), 393–399. 10.1093/ajcn/34.3.393 6782855

[eat23698-bib-0055] Whitelaw, M. , Gilbertson, H. , Lam, P. Y. , & Sawyer, S. M. (2010). Does aggressive refeeding in hospitalized adolescents with anorexia nervosa result in increased hypophosphatemia? The Journal of Adolescent Health: Official Publication of the Society for Adolescent Medicine, 46(6), 577–582. 10.1016/j.jadohealth.2009.11.207 20472215

[eat23698-bib-0056] Zipfel, S. , Löwe, B. , Reas, D. L. , Deter, H. C. , & Herzog, W. (2000). Long‐term prognosis in anorexia nervosa: Lessons from a 21‐year follow‐up study. Lancet (London, England), 355(9205), 721–722. 10.1016/S0140-6736(99)05363-5 10703806

